# Prenatal tobacco smoke exposure increases hospitalizations for bronchiolitis in infants

**DOI:** 10.1186/s12931-015-0312-5

**Published:** 2015-12-22

**Authors:** Marcello Lanari, Silvia Vandini, Fulvio Adorni, Federica Prinelli, Simona Di Santo, Michela Silvestri, Massimo Musicco, on behalf of the “Study Group of Italian Society of Neonatology on Risk Factors for RSV Hospitalization”

**Affiliations:** Pediatrics and Neonatology Unit, Imola Hospital, Via Montericco, 4, Imola, Italy; Neonatology Unit, S.Orsola-Malpighi Hospital, University of Bologna, Via Massarenti 11 40138, Bologna, Italy; Epidemiology and Biostatistics Unit, Institute of Biomedical Technologies, National Research Council Milan, Via Fratelli Cervi 93, Segrate, MI Italy; Pediatric Pulmonology and Allergy Unit, Istituto Giannina Gaslini, Genoa, Italy; Department of Neuroscience, Foundation IRCCS Santa Lucia, Via Ardeatina 306, Rome, Italy

**Keywords:** Tobacco smoke exposure, Pregnancy, Infant, Bronchiolitis, Hospitalization, Risk factor

## Abstract

**Background:**

Tobacco smoke exposure (TSE) is a worldwide health problem and it is considered a risk factor for pregnant women’s and children’s health, particularly for respiratory morbidity during the first year of life. Few significant birth cohort studies on the effect of prenatal TSE via passive and active maternal smoking on the development of severe bronchiolitis in early childhood have been carried out worldwide.

**Methods:**

From November 2009 to December 2012, newborns born at ≥33 weeks of gestational age (wGA) were recruited in a longitudinal multi-center cohort study in Italy to investigate the effects of prenatal and postnatal TSE, among other risk factors, on bronchiolitis hospitalization and/or death during the first year of life.

**Results:**

Two thousand two hundred ten newborns enrolled at birth were followed-up during their first year of life. Of these, 120 (5.4 %) were hospitalized for bronchiolitis. No enrolled infants died during the study period.

Prenatal passive TSE and maternal active smoking of more than 15 cigarettes/daily are associated to a significant increase of the risk of offspring children hospitalization for bronchiolitis, with an adjHR of 3.5 (CI 1.5–8.1) and of 1.7 (CI 1.1–2.6) respectively.

**Conclusions:**

These results confirm the detrimental effects of passive TSE and active heavy smoke during pregnancy for infants’ respiratory health, since the exposure significantly increases the risk of hospitalization for bronchiolitis in the first year of life.

**Electronic supplementary material:**

The online version of this article (doi:10.1186/s12931-015-0312-5) contains supplementary material, which is available to authorized users.

## Background

Tobacco smoke exposure (TSE) is a worldwide health problem and a risk factor for children’s health particularly for respiratory morbidity during the first years of life [1–6]. TSE during pregnancy, including both passive and active smoke, is also a well known risk factor for several complications of pregnancy and for respiratory disorders of offspring during the first months of life [1–3].

Moreover, TSE has been associated to a more severe clinical course of bronchiolitis [4-6], often requiring hospitalization. Viral bronchiolitis is a common lower respiratory tract infection in infants and often requires hospitalization in children younger than 2 years of age. It is characterized by wheezing and mucous plugging, resulting in airway obstruction [7].

Although the detrimental effects of TSE for children’s health are well known, the exposure continues to involve a large amount of infants worldwide: in 2004 about 40 % of the entire pediatric population was exposed to second-hand smoke, with 166,000 of the 5,939,000 respiratory infections and deaths due to this exposure in children younger than 5 years [8]. In several countries, smoke banning laws have been promoted with the aim to reduce the exposure in public places and subsequently protect high risk people, such as pregnant women, newborns and young infants from passive TSE. The introduction of smoke-free legislation in many countries has been related to about a ten percent decrease in preterm births (10.4 %) and in hospital admissions for asthma (10.1 %) [9].

In Italy, the estimated number of smokers is 11.6 million (7.1 million men and 4.5 million women), according to a national report considering the entire Italian population [10]. A comprehensive smoking ban was introduced in 2003 to prohibit smoking in public places, causing a 6.3 % decrease of active smokers in the Italian population [11].

In an Italian cohort study of infants younger than 2 years hospitalized for acute LRTI [12], postnatal TSE results to be a significant risk factor for hospitalization.

To our knowledge, few birth cohort studies [13–16] have been carried out that focus on the effect of prenatal TSE via passive and active maternal smoking on the development of lung diseases in childhood, which provide reports that smoke exposure in early life may increase the lung susceptibility to air pollution [16] and that prenatal TSE is related to an earlier onset of asthma. Moreover, no recent birth cohort study has investigated the effect of prenatal and early postnatal TSE on the risk of hospitalization for bronchiolitis during the first year of life.

The aim of the present study is to determine the effects of prenatal passive and active TSE and early postnatal TSE with other risk factors for hospitalization for bronchiolitis in a large cohort of preterm newborns at GA 33 weeks or more and full term newborns.

## Methods

### Study subjects

This study was promoted by the Italian Society of Neonatology with the National Research Council and involved thirty neonatology units, registering 1,000 births or more each year, in the northern, central and southern areas of Italy. The study was approved by each one of the ethical committees of the participating centres. Parents in the study provided their signed informed consent to participate in the study; details on the modalities of recruitment and of data collection have been described elsewhere [17, 18]. Briefly, all consecutive newborns at 33–34 weeks gestational age (wGA) seen in a recruitment period lasting 1 year from the first enrolment by the participating centres were enrolled. For each enrolled newborn of 33–34 wGA, two newborns of the same sex and with the nearest date of birth were enrolled: one of 35–37 wGA and one of >37 wGA. The enrolment was carried out according to GA with the aim to analyse potential differences related to GA. Exclusion criteria were: life expectancy shorter than 6 months; participation in clinical studies on pharmacological or surgical interventions; haemodynamically significant congenital heart diseases or chronic lung diseases; programmed or administered RSV prophylaxis with a humanized monoclonal antibody (palivizumab). Excluded infants were those not residing in the geographical catchment area of the enrolling neonatology unit hospital.

### Data collection

From November 1st 2009 to December 30th 2012, 2,314 newborns (1,113 females and 1,201 males) were enrolled in the study. At time of birth, parents were interviewed with a structured questionnaire on their demographics, health and socio-demographic status, living conditions and TSE. In addition, information on pregnancy, delivery and newborn conditions given by the parents were integrated, when necessary, with the information contained in the clinical record form. The same physician in each center collected the data using a standard record form. After discharge, two structured follow-up phone interviews with the child’s parents were carried out by trained interviewers. The first interview took place during the infective respiratory epidemic season (in Italy from November to March [19]), the second at the 12 month after birth. The interviews collected data on possible environmental risk conditions for bronchiolitis, including household health and crowded living conditions, daily contact with siblings or other children and day care attendance and lack of or early interruption or no breastfeeding.

### Assessment of pre- and postnatal TSE

Exposure to tobacco smoke during pregnancy was assessed by asking the mother whether she smoked during pregnancy, and if so, how many cigarettes per day and whether the father or other people smoked regularly in her presence. The information collected was then collapsed into a single variable with the following four categories for prenatal TSE: no; only passive; active with 1–15 cigarettes per day and active with 16 or more cigarettes per day.

Exposure to tobacco smoke after birth was assessed during the two follow-up interviews by asking the parents if the mother, father or other people in the household smoked inside and/or outside of the child’s living environment in the first year of life. Similarly as for prenatal TSE, the information collected was collapsed into a single variable; postnatal TSE with the following three categories: no; outside the child’s living environment and in the child’s living environment. Finally, a third variable was constructed: any tobacco smoke exposure that was comprehensive of any prenatal or postnatal exposure.

### Outcome

The main outcome of this study was hospitalization and/or death due to bronchiolitis during the first year of life, as classified by the ICD-9 code 466.1 (codifying for acute bronchiolitis) [20].

During the two follow up interviews, parents were asked about hospitalizations of the child. If the child was referred as having been hospitalized, further confirmation was obtained by retrieval of the hospital record forms or by direct contact with the physician who was responsible of the infant during hospitalization.

### Statistical analysis

The cumulative time-dependent risks of hospitalization for bronchiolitis during the first year of life of the infants were calculated with survival analysis. Relative risks were estimated as hazard ratios (HR) with 95 % Confidence Intervals (CI) calculated from the standard errors derived from Cox’s proportional hazard model [21]. Multivariate analyses were carried out to estimate the independent contribution of each considered factor to the risk of bronchiolitis.

In order to identify further potential predictors of bronchiolitis hospitalization besides exposure to tobacco smoke, we used a two-step approach. Firstly, we performed a multivariate analysis within the prenatal and the neonatal variables considered as potential risk factors for bronchiolitis (Table 1 of the Additional file 1). The variables significantly associated with the outcome (the level of statistical significance was set at *p*-value = 0.05), served for defining the exposure to pre and perinatal risk factors of bronchiolitis. Children were considered prenatally exposed (father suffering from respiratory diseases, no recourse to assisted reproductive technologies and use of corticosteroid therapy for lung maturation) and/or perinatally exposed (male sex, singleton delivery and surfactant therapy), when at least one of these significant risk factors was present. Pre and perinatal exposures were then entered in the final multivariable analysis as single dichotomous variables. Although non statistically significant in the first step of the analysis, age of the mother (continuous variable), parents’ level of education in years of completed school (both less or equal to 8 years, at least one more than 8 and less or equal to 13 years, both more than 13 years) and weeks of gestational age were considered as potential confounding variables and were entered in the final multivariable analysis.

**Table 1:** Socio-Demographic, pre-, neo-, and post- natal conditions of healthy and bronchiolitis hospitalized infants

		Hospitalization for bronchiolitis	
	Total	No	Yes
Socio demographic characteristics of parents			
Mother’s Age (y, mean ± SD)	33.6 ± 5.3	33.6 ± 5.3	33.2 ± 5.5
Parents’ Educational Level			
Primary, N. (%)	270	251 (12.0)	19 (15.8)
High, N. (%)	1521	1440 (68.9)	81 (67.5)
Graduate, N. (%)	419	399 (19.1)	20 (16.7)
Gestational age at birth			
Weeks			
33–34, N. (%)	737	683 (32.7)	54 (45.0)
35–37, N. (%)	767	726 (34.7)	41 (34.2)
> 37, N. (%)	706	681 (32.6)	25 (20.8)
Risk conditions			
Prenatal Risk	2092	1974 (94.4)	118 (98.3)
Neonatal Risk	1945	1837 (87.9)	108 (90.0)
Environmental risk conditions			
Exposure to Epidemic Season	1323	1234 (59.0)	89 (74.2)
No breastfeeding	482	440 (21.1)	42 (35.0)
Presence of siblings	900	820 (39.2)	80 (66.7)
Crowded Living Conditions	227	200 (9.6)	27 (22.5)
Day Care Attendance	357	331 (15.8)	26 (21.7)
Any smoke exposure	1043	976 (46.7)	67 (55.8)
Total	2210	2090 (94.6)	120 (5.4)

We considered also well-known environmental risk factors (RFs) of bronchiolitis as single covariates: exposure to epidemic season (defined as children living for at least one of the first 3 months of life during the calendar period between November to March); no breastfeeding (i.e. feeding of the newborn without breast milk beyond the age of 1 month); presence of siblings or children (less than 10 years old) sharing the same living environments; crowded living conditions (i.e. the presence of five or more people older than 10 years in the living environment of the newborn) and day care attendance.

Sample size was calculated by fixing a predefined precision of the estimate of the absolute risk of hospitalization and/or death for bronchiolitis induced or not by RSV during the first year of life. Assuming that the risk of hospitalization for bronchiolitis during the first year of life was about 7 % a sample size of 2,500 newborns could provide a 95 % confidence interval (95 % CI) of 6.0 to 8.1 % which is largely consistent with a random error of less than 20 %.

The level of statistical significance was set for all the analysis at *p* = 0.05.

The software package used was IBM SPSS Statistics for Windows version 21.0 (Armonk, NY, IBM Corp.).

## Results

Of the 2,314 newborns, 104 were not available for the first scheduled follow-up interview and thus the remaining 2,210 (1,150 male and 1,060 female) were considered for this analysis. All of the infants survived the entire follow-up period. One hundred and 20 newborns (5.4 %) were hospitalized for bronchiolitis during the study period, most frequently during the very early period of life (54 % within the 3 month of life). In only 31 newborns hospitalized for bronchiolitis a laboratory test was carried-out resulting positive for RSV in 26 (83 %) cases. Parental socio-demographic and infants’ characteristics are reported in Table 1 by hospitalization for bronchiolitis. Low level of parents’ education, low gestational age and all the considered prenatal, neonatal and postnatal risk conditions were more frequent in infants hospitalized for bronchiolitis. Moreover, any exposure (pre and post-natal) to tobacco smoke was more frequently registered in children who were hospitalized for bronchiolitis

The different exposures and characteristics of the infants according to exposure to tobacco smoke during the prenatal and postnatal life are reported in Table 2. TSE (both prenatal and postnatal) was more frequent in babies born to parents with only primary education. Mothers smoking more than 15 cigarettes per day during pregnancy more frequently had newborns with low gestational ages. Maternal smoking of more than 15 cigarettes per day during pregnancy was associated with an increase of non-breastfeeding of the newborn. Presence of siblings was associated with TSE both of the mother during pregnancy and of the newborn in postnatal life period. Also, living in crowded living conditions was more frequent for newborns exposed to smoke in prenatal and postnatal periods.

**Table 2:** Association of infants’ pre- and post- natal TSE with socio-demographic, pre-, neo-, post- natal conditions and bronchiolitis hospitalization

		Prenatal smoking exposure				Postnatal smoking exposure		
			Passive	Active maternal smoking				
Newborn and parents’ characteristics	Total	None	maternal smoking	≤15 cig/day	>15 cig/day	None	Out of living environment	In living environment
Socio demographic characteristics of parents								
Mother’s Age	33.6 ± 5.3	33.9 ± 5.2	32.9 ± 5.1	32.5 ± 5.8	34.0 ± 5.5	34.1 ± 5.2	32.8 ± 5.3	33.5 ± 5.5
(y, mean ± SD)								
Parents’ Educational Level								
Primary, N. (%)	270	159 (9.7)	50 (14.9)	50 (26.3)	11 (27.5)	115 (8.8)	136 (17.1)	19 (17.6)
High, N. (%)	1521	1122 (68.2)	243 (72.3)	128 (67.4)	28 (70.0)	885 (67.7)	564 (71.0)	72 (66.7)
Graduate, N. (%)	419	363 (22.1)	43 (12.8)	12 (6.3)	1 (2.5)	308 (23.5)	94 (11.8)	17 (15.7)
Gestational Age at birth								
Weeks								
33–34, N. (%)	737	559 (34.0)	102 (30.4)	60 (31.6)	16 (40.0)	427 (32.6)	273 (34.4)	37 (34.3)
35–37, N. (%)	767	560 (34.1)	114 (33.9)	81 (42.6)	12 (30.0)	447 (34.2)	279 (35.1)	41 (38.0)
> 37, N. (%)	706	525 (31.9)	120 (35.7)	49 (25.8)	12 (30.0)	434 (33.2)	242 (30.5)	30 (27.8)
Risk conditions								
Prenatal Risk	2092	1551 (94.3)	323 (96.1)	180 (94.7)	38 (95.0)	1231 (94.1)	760 (95.7)	101 (93.5)
Neonatal Risk	1945	1445 (88.5)	299 (89.0)	165 (86.8)	36 (90)	1137 (86.9)	712 (89.7)	96 (88.9)
Environmental risk conditions								
Exposure to Epidemic Season	1323	998 (60.7)	196 (58.3)	108 (56.8)	21 (52.5)	790 (60.4)	455 (57.3)	78 (72.2)
No Breastfeeding	482	352 (21.4)	70 (20.8)	45 (23.7)	15 (37.5)	289 (22.1)	169 (21.3)	24 (22.2)
Siblings	900	644 (39.2)	145 (43.2)	89 (46.8)	22 (55.0)	520 (39.8)	330 (41.6)	50 (46.3)
Crowded Living	227	160 (9.7)	35 (10.4)	25 (13.2)	7 (17.5)	141 (10.8)	68 (8.6)	18 (16.7)
Day Care Atten dance Attendance	357	265 (16.1)	43 (12.8)	43 (22.6)	6 (15.0)	221 (16.9)	117 (14.7)	19 (17.6)
Hospitalizations for bronchiolitis (%)	120 (5.4)	77 (4.7)	26 (7.7)	11 (5.8)	6 (15.0)	65 (5.0)	47 (5.9)	8 (7.4)
TOTAL	2210	1644	336	190	40	1308	794	108

**Fig. 1:**
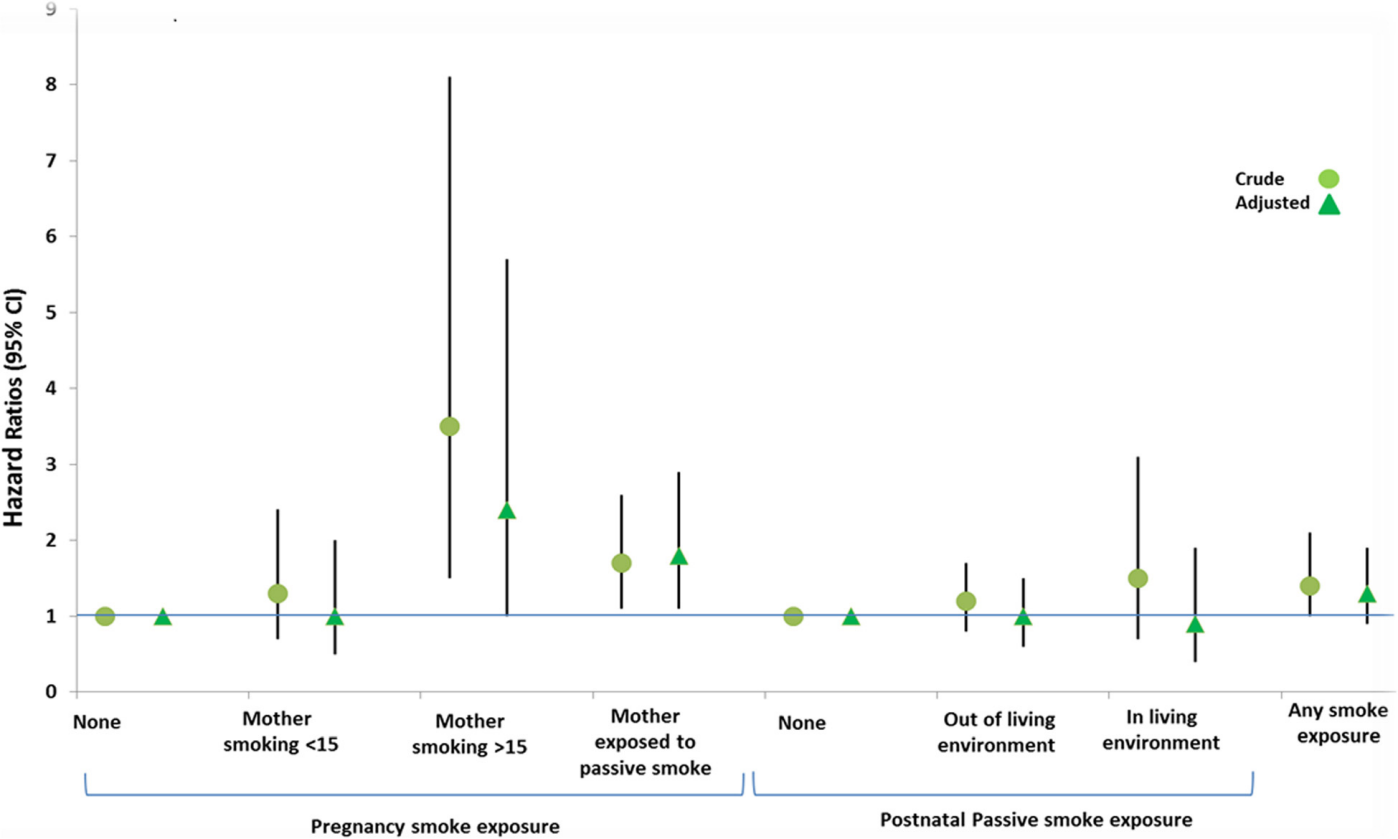
Crude and Adjusted Hazard Ratios (HRs) for Prenatal and Postnatal Tobacco Smoke Exposure for bronchiolitis Hospitalization in the First Year of Life. Adjusted for mother’s age (y), parents’ educational level, wGA, prenatal risk conditions, neonatal risk conditions, exposure to epidemic season, breastfeeding, siblings, crowded living, day care attendance

Table 3 and Fig. 1 describe the association of tobacco smoke exposure and all considered risk factors, with the risk of hospitalization for bronchiolitis during the first year of life. At univariate analysis, the exposure to any tobacco smoke was associated with a 40 % increased risk of hospitalization (HR = 1.4, 95 % CI 1.0–2.1). When accounting for all the other considered variables in multivariable analysis, the risk was still increased however lost statistical significance (HR 1.3, 95 % CI 0.9–1.9). Moreover, when considering exposure to prenatal tobacco smoke, having a mother smoking more than 15 cigarettes every day or a mother exposed to second hand smoke were associated with a significant risk increase of 3.5 (CI 1.5–8.1) and of 1.7 (1.1–2. 6), respectively. In multivariable analysis these risks remained substantially equal in size and direction. As far as tobacco exposure in postnatal period is concerned, only small non-significant risk increases were observed in univariate analysis that were no longer present in multivariable analysis. Among the other considered variables, the most remarkable results from the multivariable analysis were a negative association with mother’s age and with increasing gestational age at birth, positive significant associations with all the environmental risk conditions and finally, no significant association with parent’s educational level and with prenatal or perinatal risk conditions.

**Table 3:** Crude and adjusted hazard ratios for risk factors for hospitalization for lower respiratory tract infections in the first year of life

	Hazard ratios (95 % CI)			
	Crude HR	*p*-value	Adjusted HR	*p*-value
Tobacco smoke exposure				
Any	1.4 (1.0–2.1)	0.050	1.3 (0.9–1.9)	0.131
Prenatal				
None	1		1	
Mother smoking ≤15 cigarette	1.3 (0.7–2.4)	0.476	1.0 (0.5–2.0)	0.964
Mother smoking >15 cigarette	3.5 (1.5–8.1)	0.003	2.4 (1.0–5.7)	0.052
Mother exposed to passive smoke	1.7 (1.1–2.6)	0.021	1.8 (1.1–2.9)	0.024
Postnatal				
None	1		1	
Out of living environment	1.2 (0.8–1.8)	0.343	1.0 (0.6–1.5)	0.869
In living environment	1.5 (0.7–3.1)	0.275	0.9 (0.4–1.9)	0.707
Socio demographic characteristics of parents				
Mother’s Age (y)	0.9 (0.8–1.1)	0.344	0.8 (0.7–1.0)	0.055
Parents’ Educational Level				
Primary	1		1	
High	0.7 (0.5–1.2)	0.256	1.0 (0.6–1.7)	0.956
Graduate	0.7 (0.4–1.3)	0.215	1.1 (0.6–2.3)	0.727
Gestational Age at birth				
Weeks				
33–34	1		1	
35–37	0.7 (0.5–1.1)	0.114	0.7 (0.4–1.0)	0.046
> 37	0.5 (0.3–0.8)	0.002	0.5 (0.3–0.8)	0.002
Risk conditions				
Prenatal	3.4 (0.8–13.8)	0.086	2.3 (0.6–9.7)	0.242
Perinatal	1.2 (0.7–2.2)	0.497	1.4 (0.7–2.5)	0.315
Environmental risk conditions				
Exposure to Epidemic Season	2.0 (1.3–3.0)	0.001	1.9 (1.2–2.8)	0.003
Breastfeeding	2.0 (1.4–2.9)	0.000	1.8 (1.2–2.7)	0.003
Siblings	3.0 (2.1–4.4)	0.000	3.2 (2.2–4.8)	0.000
Crowded living Conditions	2.6 (1.7–4.1)	0.000	2.5 (1.6–3.9)	0.000
Day care attendance	1.4 (0.9–2.2)	0.113	1.7 (1.1–2.7)	0.015

## Discussion

The results of this study demonstrate that passive TSE and heavy active smoke exposure during pregnancy increases the risk of hospitalization for bronchiolitis during the first year of life, and in particular, during the first 3 months of life, while postnatal TSE does not significantly increase the risk in our cohort. These results could be explained by the fact that passive exposure is less preventable than active smoking, which is often interrupted during pregnancy. Our results suggest that the detrimental effect on lung development and subsequent higher risk for hospitalization may depend mostly on prenatal exposure rather than to postnatal TSE. This could be due to the fact that most mothers quit smoking during the first trimester, however by this time, the pulmonary system reaches its full development and therefore deleterious influences could play a major role.

The detrimental effects of TSE during pregnancy on fetal growth and lung development have been widely studied. Fetal TSE increases the risk for intrauterine growth retardation, prematurity and impaired lung development through placental vascular damage induced by cotinine, which causes placental insufficiency and nutritional deprivation that may interfere with growth and lung maturation [22] and through epigenetic mechanisms. [23–25] These mechanisms are determined by several metabolites derived from tobacco smoke that have low molecular weight and are able to cross the placenta and induce oxidative damage, mitochondrial dysfunction and chronic hypoxia leading to genetic modifications that affect fetal growth and development [26, 27]. Appropriate development of fetal lung requires adequate nutritional intake and placental blood flow as well as the precisely timed gene expression that is, in part, dependent from the extensive combination of DNA methylation and histone modifications. Thus the interference with the normal epigenetic modifications during fetal life can alter gene transcription and may alter lung growth and function [24, 27].

Recently, some authors have observed that fetal growth is also influenced by maternal grandmother smoking habits during pregnancy [28, 29] of non-smoking mothers, likewise, the attitude toward smoking of the grandmother is associated to an elevated risk of asthma in children [30, 31]. The mechanism hypothesized for these effects include a cascade of metabolic knock-on effects involving initial somatic metabolic *in utero* “programming” of the mother and a direct effect of grandmaternal smoking on oogenesis of her own daughter when she was a fetus [32, 33]. It is possible that by modifying DNA methylation patterns in the fetal oocytes, tobacco-derived metabolites may interfere with both immune function and xenobiotic detoxification mechanisms in the offspring, resulting in an increased susceptibility to respiratory diseases affecting the subsequent generation.

In our study, we found that active smoking during pregnancy has an effect on the risk for bronchiolitis hospitalization that is dose-dependent. We defined heavy smoking mothers who smoked more than 15 cigarettes/day, as previously done in another study [34], with the aim to emphasize the effect of a discrete number of cigarettes. The dose-dependency of active smoking during pregnancy was observed also for growth restriction in previous studies [27, 35]. This result suggests that a reduction in the number of smoked cigarettes, particularly in heavy smokers, may reduce the risk for hospitalization in infants. Moreover, active smoking during pregnancy was associated to lower GA, confirming the effect of tobacco smoke on the risk of preterm birth; the effect of active smoke however was confirmed by the multivariate analysis, establishing that it is independent from GA.

To date, only a few studies have distinguished between prenatal and postnatal TSE and their consequences on respiratory health in early life [36, 37].

The study by Jaakkola et al. [38] demonstrates the strongest adverse effects of tobacco smoke on the lower respiratory tract when smoking takes place during pregnancy. In prenatally exposed children, postnatal exposure from either parent did not significantly increase the occurrence of the respiratory health outcomes analysed by Fuentes-Leonarte et al. [39].

TSE in infants may cause bronchial hyper-reactivity and direct toxic and irritant effects on the lungs and the airways [23]. TSE may increase the susceptibility to pathogens due to impaired protective mechanisms of the airways and bronchial tree, such as mucociliary clearance [4]. Infants of women smokers are more likely to have diminished lung function soon after birth, which could contribute to the development of acute respiratory outcomes such as infections as its concomitant symptoms. [40] We found that postnatal TSE during the first year of life was associated to a slightly higher hospitalization rate without reaching the statistical significance. This limited time span may underestimate the total effect of exposure since assessing respiratory tract infections at older ages might show stronger effects. However, our results are consistent with those obtained by Duijts et al. [41] which analysed a prenatally enrolled birth-cohort and showed weak evidence for an association of maternal smoking in the early postnatal period with bronchiolitis in infants in the first 6 months of life [41].

The strength of the present study is the prospective analysis of a large birth cohort and the inclusion of a large number of other variables that may interact with TSE in the increase of bronchiolitis severity and hospitalization.

One of the limits of the present study is that TSE was assessed on subjective parent report, however other studies reported good agreement between self-reported exposure and biochemical markers dosed in the household environment and in urine samples [42–44].. Another limitation is related to the fact that we considered only hospitalizations for bronchiolitis and therefore factors related to both the propensity to recur to hospitalization and to TSE, such as socio-cultural conditions of the parents, which may act as confounders of our results. Nevertheless, we hold the conviction that the universal coverage of the Italian National Health Service, which is free for every Italian citizen, guarantees virtually equal access to hospital medical care to everyone in cases concerning severe bronchiolitis. In our cohort study, heavy active smoking during pregnancy was associated to lack of breastfeeding and to crowded living conditions, and it was more frequent if parents had only primary level education. This data could be explained by the lower attention paid by a subgroup of women during pregnancy to campaigns promoting prenatal and neonatal health. This is an interesting note as it could be useful to identify pregnant women that may require an implementation of counselling to promote breastfeeding and to improve the overall living conditions of their newborns.

Our study supports the evidence of the strongest effects of TSE during pregnancy and supports that smoking cessation campaigns should target all family and household members as well as colleagues in workplaces of non-smoking expecting mothers. Moreover, the knowledge of the detrimental effects of TSE on infants could increase awareness regarding the importance of reducing second-hand smoke exposure, not only in public places but in private households as well as.

Health professionals play a pivotal role in promoting the cessation of active smoking and passive smoke exposure in pregnant women and more should be done: a recent Australian study [45] reported that less than half pregnant women were discourage to stop smoking by healthcare professionals.

Data obtained in the present study could be used by practitioners, obstetricians, neonatologists, pediatricians, midwives and other healthcare professionals in order to improve the information provided during counselling to pregnant women and new parents about the risks of TSE on their offspring. Extended follow up of this cohort of children will allow us to establish whether these exposures are important predictors of asthma in later childhood and a possible cause of permanent impairment of pulmonary function. Moreover, further investigations could be useful to clarify the effect of TSE in pediatric patients with other well known risk factors for hospitalization for bronchiolitis (i.e., prematurity, congenital heart disease, chronic lung disease, immunodeficiency, neuromuscular disease.

## Conclusions

Bronchiolitis is a major cause of hospitalization for infants and passive and active maternal TSE during pregnancy seems to be significant RFs that can be modified. According to the tendencies found in this study, the elimination of smoking during pregnancy and the avoidance of second-hand TSE in the environment where pregnant women live are mandatory in order to reduce morbidity of newborns and young infants, also in the presence of other non-modifiable RFs for bronchiolitis. Moreover, since women are more likely to quit smoking during pregnancy than at any other time, there are efforts to increase motivation and help women who are planning to conceive to stop smoking at the procreative phase of their life or during pregnancy. Finally, studies on respiratory tract infections in infants at a later age are necessary to examine the long-term effects of maternal TSE during and after pregnancy. The results of the present study will be used by the Italian Neonatology Society, that supported the study, to improve counselling to families and pregnant women to reduce tobacco smoke exposure.

## Additional file

Additional file 1:Table S1. Risk of bronchiolitis according to prenatal and neonatal risk conditions of newborns (DOCX 26 kb)

## Abbreviations

AdjHR: Adjusted hazard ratio
CI: Confidence interval
GA: Gestational age
HR: Hazard ratio
LRT: Lower respiratory tract infections
Rfs: Risk factors
RSV: Respiratory syncytial virus
TSE: Tobacco smoke exposure
wGA: Weeks of gestational age
