# Supplementary material for: Prenatal tobacco smoke exposure increases hospitalizations for bronchiolitis in infants
Source: Respir Res. 2015 Dec 22;16:152. doi: 10.1186/s12931-015-0312-5 (PMC4699376; doi:10.1186/s12931-015-0312-5)
Supplement: Additional file 1:Table S1. — Risk of bronchiolitis according to prenatal and neonatal risk conditions of newborns (DOCX 26 kb) [file 12931_2015_312_MOESM1_ESM.docx]

**SUPPLEMENTARY FILE**

**Table 1. Risk of bronchiolitis according to prenatal and neonatal risk conditions of newborns**

| ***Prenatal risk conditions*** | p-value | HR (CI 95.0%) | | |
| --- | --- | --- | --- | --- |
| **Father’s respiratory diseases*** | **.008** | **3. 4 (1.4-8. 3)** | | |
| **Assisted reproductive technologies** | **.004** | **0.3 (0.1-0.7)** | | |
| **Use of corticosteroids for lung maturation** | **.000** | **2.1 (1.4-3.0)** | | |
| Mother’ s diseases potentially conditioning the pregnancy outcome** | .176 | 1.31 (0.89-1.92) | | |
| IUGR | .329 | .68 (0.31-1.47) | | |
| Mother’s respiratory diseases* | .357 | 1.73 (0.54-5.55) | | |
| Father education (≤ 8 years) | .290 | 1.24 (0.83-1.87) | | |
| Mother education (≤ 8 years) | .666 | 1.10 (0.71-1.72) | | |
|  |  |  | | |
| ***Neonatal risk conditions*** |  |  |  |  |
| **Male sex** | **.015** | **1.6 (1.1-2.3)** | | |
| **singleton delivery** | **.006** | **2.0 (1.20-3.2)** | | |
| **surfactant therapy** | **.036** | **2.3 (1.1-4.9)** | | |
| Week of gestational age at delivery |  |  |  |  |
| 33-34 | .369 |  | 1** |  |
| 35-37 | .489 | 0.9 (0.6-1.3) | | |
| 37+ | .158 | 0.60 (0.3-1. 2) | | |
| Cesarean section delivery | .100 | 1.5 (0.9-2.3) | | |
| neonatal hospitalization | .225 | 1. 4 (0.8-2.3) | | |
| neonatal resuscitation | .818 | 1.1 (0.7-1.7) | | |
| neonatal respiratory diseases | .932 | 1.0 (0.6-1.7) | | |
| antibiotics therapy during hospitalization | .715 | 1.1 (0.6-2.3) | | |
| APGAR 5’ score < 8 | .509 | 0.8 (0.3-1.7) | | |

* History of BPCO, asthma and/or wheezing, recurrent bronchitis.

** Defined on judgment of the recruiting physician (mostly diabetes and pre-eclampsia)

*** Reference category
